# Hair of Women

**Published:** 1874-06

**Authors:** 


### HAIR OF WOMEN.

THE longest and most luxurious hair obtained by hair merchants for the Paris and London markets is taken from the heads of girls who keep the hair enveloped in a handkerchief from the day they sell it to the time when the next crop is ready for the market; these are peasant girls of a low grade in society and untidy in all their habits. Indian women have long, thick, unwhitened hair to a good old age, in very many cases, and yet they wear their hair in such an unkempt condition that it is in many cases a matted mass of inextricable tangles. Luxuriant hair is to some extent an inherited quality, and, to a considerable extent, it belongs to a healthy constitution. But the hair can be cultivated, it can be made healthy and preserved in its natural color until fifty, with proper care and attention.

The first point is a clean scalp. The second entire freedom, not a pin, or a comb, or a twist, or a braid about it. Simply combed back from the forehead and hanging down behind, a ribbon confining it just behind and under the ears so as to prevent it from coming forward over the cheeks. The first point is to have it as freely exposed to the air, day and night, as possible, because the perspiration escapes in the form of steam and carries away the extra heat, and thus keeps the scalp healthfully cool and more cleanly than if confined with a kerchief or impervious material. Whether covered or exposed to the air, as in squaws, there is one element alike tending to the preservation of the hair—it is undisturbed, not rendered harsh and brittle bythe use of iron hair pins; India rubber pins are greatly better, because smoother; the hair is not dragged by its roots, and against its natural direction, nor is it tortured by twisting and plaiting and frizzing, all which are pernicious, by inflaming the hair roots and loosening them, and interfering with the healthful flow of blood to the parts for purposes of strength and growth. Combing the hair does good when properly performed.

The comb should be slowly and carefully drawn through, so as not to break a hair, or drag upon its root; as soon as the comb leaves the scalp, the fingers of the other hand should catch hold of the hair behind the comb, so as not to drag a single hair from the scalp. If there is any confinement of the back hair, it should not be done by a string, and far less with hair pins, but coil it loosely on the back of the neck and keep in position with a tortoise shell comb. The more free the hair is the better; attend to this, and to one other fact—never pretend to dress it with anything other than pure water; it is the only healthful dressing; an oil or pomade was never sold that did not injure the hair irrecoverably, if used as a habit. This cannot be too strongly impressed on the reader's mind. All hair brushes should be soft, so as not to inflame the scalp by scratching it. Any kind of night cap is pernicious, however light or however loosely worn. Oiled silk night caps are especially hurtful, for they confine the perspiration to the scalp and keep it in a sodden condition, beside preventing the escape of exhalations of waste and dead material.

If the hair were allowed to hang freely at night, it would be better ventilated—and abundant ventilation is its life. If at times the hair must be confined, let it be done with very fine net work, having large meshes.

